# LIN28 Is Involved in Glioma Carcinogenesis and Predicts Outcomes of Glioblastoma Multiforme Patients

**DOI:** 10.1371/journal.pone.0086446

**Published:** 2014-01-24

**Authors:** Rong Qin, Jingxu Zhou, Chao Chen, Tao Xu, Yong Yan, Yushui Ma, Zongli Zheng, Yiping Shen, Yicheng Lu, Da Fu, Juxiang Chen

**Affiliations:** 1 Department of Neurosurgery, Shanghai Institute of Neurosurgery, Changzheng Hospital, Second Military Medical University, Shanghai, China; 2 Department of Neurosurgery, the 101^st^ hospital of PLA, Wuxi, Jiangsu Province, China; 3 Key Laboratory of Stem Cell Biology, Institute of Health Sciences, Shanghai Jiao Tong University School of Medicine & Shanghai Institutes for Biological Sciences, Chinese Academy of Sciences, Shanghai, China; 4 Department of Pathology, Massachusetts General Hospital, Harvard Medical School, Boston, Massachusetts, United States of America; 5 Department of Laboratory Medicine, Children's Hospital Boston, Harvard Medical School, Boston, Massachusetts, United States of America; Medical Center at Seattle, United States of America

## Abstract

LIN28, an evolutionarily conversed RNA binding protein which can bind to the terminal loops of let-7 family microRNA precursors and block their processing to maturation, is highly expressed in several subsets of tumors that carry poor prognoses, such as ovarian carcinoma, hepatocellular carcinoma, colon carcinoma and germ cell carcinoma. However, there has been no study on the expression of LIN28 in glioma tissues or their importance as a prognostic predictor of glioma patients. This study aimed to examine the expression of LIN28 in glioma and correlate the results to patient outcome. We found that LIN28 expression was significantly higher in the group of patients with a poor prognosis compared to patients with a good prognosis by gene microarray. Log-rank analysis showed patients with higher LIN28 expression level in tumor had a shorter progression-free survival and overall survival times compared to those with lower LIN28 expression level. Similar results were also obtained from the tissue microarray analysis. Univariate and multivariate analyses showed high LIN28 expression was an independent prognostic factor for a shorter progression-free survival and overall survival in GBM patients. Furthermore *in vitro* experiments showed that down-regulation of LIN28 in U251 and U373 cells caused cell cycle arrest in the G1 phase, delayed cell proliferation, increased apoptosis, and resulted in fewer colonies compared to controls. Summarily, our data provides a potential target for cancer therapy as an approach to overcome the poor options currently available for GBM patients.

## Introduction

Gliomas are the most common primary brain tumor in adults, with an incidence rate of approximately five per 100,000 person-years annually worldwide. Approximately 70% of gliomas are malignant gliomas, and the most frequent and lethal cancers originate in the central nervous system (CNS) with a high recurrence and mortality rate [1]. The 5-year survival rate is approximately 20% for patients with glioma, but <3% for patients with glioblastoma multiforme (GBM), the most biologically aggressive subtype of gliomas. Despite aggressive surgery, radiation, and chemotherapeutic options, the life expectancy of patients with GBM is still poor with a median overall survival of approximately 12–15 months after diagnosis [2].

Substantial efforts have been taken to identify molecular markers and therapeutic targets that could help to achieve a better prognosis. Several candidate genes, such as EGFR [3], SOX2 [4], and VEGF [5], have been implicated in the oncogenesis or progression of GBM. These genes could play important roles in the treatment of this severe disease. New therapeutics against these targets have potential utility as effective clinical treatments. Thus, a better understanding of the mechanisms involved in regulating tumor growth requires the identification of novel genes associated with glioma.

LIN28 is an evolutionarily conversed RNA binding protein that can bind to the terminal loops of let-7 family microRNA (miRNA) precursors and block their processing to maturation [6]–[8]. Several oncogenes are known to be targets of the let-7 miRNA family, including Ras [9], c-Myc [10], and Hmga2 [11], and the repression of let-7 has been linked to several types of tumor, such as lung [9], [10], breast [12], and ovarian [13]–[15] cancer. It has been reported that LIN28 is highly expressed in several subsets of tumors that carry poor prognoses, such as ovarian carcinoma [13]–[15], hepatocellular carcinoma [16]–[18], colon carcinoma [19], [20], and germ cell carcinoma [21]–[23]. Based on these observations, LIN28 has been shown to be functional in the post-transcriptional regulation of the let-7 miRNA family and is postulated to be oncogenic through repression of let-7 family miRNAs as well as depression of let-7 targets. However, whether LIN28 is related to the carcinogenesis of glioma and the mechanism responsible are currently unclear.

Based on the complete sequencing of the human genome as well as several high-throughput genomic technologies, The Cancer Genome Atlas (TCGA) has defined three main pathways involved in GBM: the RTK/RAS/PI3K signaling pathway as well as the p53 and RB tumor suppressor pathways. The frequencies of somatic alterations in these pathways have been shown to be 88%, 87%, and 78%, respectively [24]. Previous studies have demonstrated that several upstream genes involved in these pathways, including Ras, ARF and CDK4, are associated with LIN28 [9], [25]–[27]. The purported link between LIN28 and glioma was even highlighted by the recent identification of the role of Let-7 miRNA in GBM: let-7 miRNA can reduce the *in vitro* proliferation and migration of GBM cell lines and reduce the size of xenograft tumors in nude mice [28]. However, the effects of LIN28 on the prognosis of glioma patients remain unknown.

In this study we used gene and tissue microarrays to detect the correlation between the LIN28 expression level and prognosis of glioma patients, and then performed cytological experiments to define the role of LIN28 in glioma tumorigenesis. We found that the expression of the *LIN28* gene was significantly different between the patient group with a good prognosis and the group with a poor prognosis, indicating that LIN28 may be a predictor of survival in glioma patients. The cytological experiments further illustrated the role of LIN28 in the proliferation of glioma cells.

## Materials and Methods

### Tissue Samples and Ethics Statement

The study protocol and acquisition of tissue specimens were approved by the Specialty Committee on Ethics of Biomedicine Research, Second Military Medical University, and complied with the National Regulations on the Use of Clinical Samples in China. Glioma tissue specimens were obtained from archived tissue samples from patients who underwent surgical treatment at Changzheng Hospital during the period from January 1999 to December 2010. These patients or their legal guardian provided written informed consent to the surgical procedures and gave permission to use resected tissue specimens for research purposes. A diagnosis of glioma was confirmed pathologically by two independent, experienced pathologists.

### Gene Expression Microarray

For gene expression microarray analysis, tumor tissue from 17 glioma patients was assessed on an Affymetrix Array platform (Santa Clara, CA). The demographic and clinicopathological characteristics of the 17 glioma patients are shown in Table 1. The sample preparation and microarray hybridization were performed based on the manufacturer’s standard protocols. Briefly, 1 µg of total RNA from each sample was amplified and transcribed into fluorescent cRNA using the manufacturer’s labeling protocol. The labeled cRNAs were hybridized onto the Affymetrix U133 plus 2.0. After washing the slides, the arrays were scanned by GeneChip® Scanner 3000. The data from the experiments were divided into “high” (≥1) and “low” (<1) describing the expression level of LIN28. Differentially expressed genes were identified through fold change filtering.

**Table 1:** Demographic and clinicopathological characteristics of the patients (n = 17).

Characteristics	Value	Lin28 Expression Low	Lin28 Expression High
**Number of Patient**	17	12	5
**Age(years)**			
Mean ± S.D. (Median)	41.00±11.64 (41)		
≤50	13(76.5%)	12	1
>50	4(23.5%)	0	4
**Gender**			
Male	13(76.5%)	9	4
Female	4(23.5%)	3	1
**Seizure**			
Present	4(23.5%)	3	1
Absent	13(76.5%)	9	4
**IICP**			
Present	9(52.9%)	5	4
Absent	8(47.1%)	7	1
**Cystic degeneration**			
Present	6(35.3%)	3	3
Absent	11(64.7%)	9	2
**Necrosis on MRI**			
Present	6(35.3%)	2	4
Absent	11(64.7%)	10	1
**Border on MRI**			
Clear	5(29.4%)	5	0
Unclear	12(70.6%)	7	5
**MTD(cm)**			
Mean ± S.D.(Median)	4.58±1.31 (4.24)		
<5	11(64.7%)	8	3
≥5	6(35.3%)	4	2
**Resection degree**			
Gross total resection	14(82.4%)	10	4
Subtotal resection	3(17.6%)	2	1
**Tumor grade and pathological category**			
WHO Grade I	2(11.8%)		
Pilocytic astrocytoma	2	2	0
WHO Grade II	9(52.9%)		
Astrocytoma	4	4	0
Oligodendroglioma	5	3	2
WHO Grade III	2(11.8%)		
Anaplastic astrocytoma	2	1	1
WHO Grade IV	4(23.5%)		
GBM	4	2	2
**Adjuvant Chemotherapy**			
Present	13(76.5%)	10	3
Absent	4(23.5%)	2	2
**Adjuvant Radiotherapy**			
Present	10(58.8%)	8	2
Absent	7(41.2%)	4	3
**Survival status**			
Alive	10(58.8%)	9	1
Dead	7(41.2%)	3	4

Abbreviations: IICP, increased intracranial pressure; MTD, mean tumor diameter; GBM, glioblastoma multiforme.

Hierarchical clustering was performed using the multiple experiment viewer (MeV) 4.7.1 software (http://www.tm4.org/mev/). Gene ontology (GO) analysis and pathway analysis were performed using the standard enrichment computation method. Differentially regulated genes are represented in the links predicted using STRING (http://string.embl.de/).

### Tissue Microarray Construction and Immunohistochemistry

Paraffin-embedded tissues were acquired from 90 GBM patients. The demographic and clinicopathological characteristics of the patients are shown in Table 2. Tissue microarrays of these sample specimens were constructed as previously described [29]. Immunohistochemical staining using a polyclonal anti-LIN28 antibody (Abcam, San Diego, CA) was performed according to the manufacturer’s instructions. The intensity and extent of staining were evaluated by two independent pathologists blinded to the clinicopathological data of the patients. Staining intensity in the cytoplasm was graded using a scale from 0 to 3 (0, no immunostaining; 1, light brown color; 2, medium brown color, and 3, dark brown color). The percentage of positively stained cells was scored as follows: 0, no staining; 1, ≤50% of the tumor cells; 2, 50–90% of the tumor cells; 3, >90% of the tumor cells. The final score, regarded as the expression level of LIN28, was the product of intensity and percentage scores, and was classified as follows: strong (+++, final score >6), moderate (++, final score = 4–6), weak (+, final score = 1–3), or null (−, final score = 0). For analysis, LIN28 expression was divided into “high” (++ and +++) and “low” (+ and −). All discrepancies in scoring were reviewed and a consensus was reached between the two pathologists.

**Table 2:** Demographic and clinicopathological characteristics of the patients(n = 90).

Characteristics	Value			Lin28 Expression Low	Lin28 Expression High
**Number of Patient**	90			25	65
**Age(years)**					
Mean ± S.D. (Median)	50.82±16.23 (54)				
≤50	32(35.6%)			11	21
>50	58(64.4%)			14	44
**Gender**					
Male	61(67.8%)			16	45
Female	29(32.2%)			9	20
**Seizure**					
Present	11(12.2%)			3	8
Absent	79(87.8%)			22	57
**IICP**					
Present	39(43.3%)			10	29
Absent	51(56.7%)			15	36
**Cystic degeneration**					
Present	19(21.1%)			9	10
Absent	67(74.4%)			14	53
Unknown	4(4.4%)			2	2
**Necrosis on MRI**					
Present	15(83.3%)			7	8
Absent	75(16.7%)			18	57
**Border on MRI**					
Clear	17(18.9%)			4	13
Unclear	41(45.6%)			12	29
Unknown	32(35.5%)			9	23
**MTD(cm)**					
Mean ± S.D.(Median)	4.35±1.23 (4.64)				
<5	37(41.1%)			12	25
≥5	52(57.8%)			12	40
Unknown	1(1.1%)			1	0
**Resection degree**					
Gross total resection	68(75.6%)			19	49
Subtotal resection	19(21.1%)			5	14
Partial resection	3(3.3%)			1	2
**Adjuvant Chemotherapy**					
Present		64(71.1%)		19	45
Absent		26(28.9%)		6	20
**Adjuvant Radiotherapy**					
Present		63(70.0%)		21	42
Absent		27(30.0%)		4	23
**Survival status**					
Alive		5(5.6%)		4	1
Dead		85(94.4%)		21	64

Abbreviations: IICP, increased intracranial pressure; MTD, mean tumor diameter.

### Cell Culture

The human glioblastoma cell lines U251 and U373 were obtained from the American Type Culture Collection (ATCC) and maintained as subconfluent monolayers at 37°C and 5% CO_2_ in Dulbecco’s modified Eagle’s medium/Ham’s F-12 nutrient mixture (DMEM/F-12), containing 15 mM HEPES and 2.5 mM L-glutamine. The medium was supplemented with 5% heat inactivated equine serum, 500 ng/ml hydrocortisone, 21.5 ng/ml epidermal growth factor (EGF), 10 µg/ml insulin, 100 ng/ml cholera toxin, and 10 µg/ml gentamicin.

A target sequence for the small hairpin RNA (shRNA) for LIN28 was used (5′-CTGTAACGTTGCGAATGGTAT-3′). A retrovirus expressing shRNA specific to LIN28 was used to infect U251 and U373 cells. Briefly, a retroviral vector containing a specific shRNA, pHCMV-G, or pCMV-dR8.9 were co-transfected into 293T cells, and the viral supernatants were collected to infect U251 and U373 cells. Single clonal cells were then selected, expanded, and screened for the down-regulation of LIN28.

### Quantitative RT-PCR (qRT-PCR)

To confirm the expression of LIN28 in U251 and U373 cells, the reactions were conducted on an iCycler iQ (BioRad). Each 25 µl PCR reaction included SYBR Green Supermix (BioRad) with 0.4 mM each of dNTP, 50 U/ml iTaq DNA polymerase, 6 mM Mg^2+^ SYBR Green I, and 20 nM fluoroscein. The reaction mixture also contained cDNA from U251 and U373 cells and the forward and reverse primers specific for the gene of interest. The efficiency of each primer set was calculated using a standard curve. Dilutions of cDNA ranging from 0.37–30 ng were prepared and the cycle threshold (Ct) values for the reactions were plotted against the cDNA concentrations. The comparative cycle threshold method (2^−ΔΔCt^) was used to quantify gene expression. Fold-change values represent an average of three separate experiments. β-actin served as the internal control in each qRT-PCR reaction. All measurements were performed in triplicate. The sequences of the primer pairs were as follows: LIN28 5′-GGAATCGTGGTCTCTCACTCG-3′ and 5′-GTGTGATGTCCGGACTGTCAT-3′; β-actin 5′-AGCGAGCATCCCCCAAAGTT-3′ and 5′- GGGCACGAAGGCTCATCATT-3′.

### Western Blot

Cells were lysed by vortexing on ice in 50 mM Tris (pH 7.4), 150 mM NaCl, 1 mM EDTA, 1% Triton X-100, 1 mM PMSF, and 1% protease inhibitor cocktail (Sigma). Protein concentrations were determined using the Bradford method (BioRad). Total cellular protein (50 µg) was separated on SDS-polyacrylamide gels and then transferred to polyvinyldifluoride (PVDF) membranes (Millipore). For immunodetection, the membranes were incubated in blocking buffer (5% non-fat dry milk dissolved in Tris-buffered saline solution with 0.05% Tween 20) for 60 min at room temperature to block all non-specific binding sites. The membranes were then incubated with the primary antibody diluted in blocking buffer for 60 min at room temperature. After anti-LIN28 (1∶500) or anti-GAPDH (1∶1000) antibody (Abcom, Cambridge, UK) incubation the membranes were washed in Tris-buffered saline-Tween (TBS-T). The membranes were then incubated with a goat anti-mouse horseradish peroxidase-conjugated secondary antibody (1∶20,000, Abcom, Cambridge, UK) for 60 min at room temperature before being washed again in TBS-T. Proteins were visualized with the enhanced chemiluminescence plus (ECL+) system (Amersham) on a FluorChem 8900 imaging system (Alpha Innotech). All antibodies were obtained from Santa Cruz Biotechnology, Inc. (Santa Cruz, CA). GAPDH was used as a loading control.

### Cell Cycle Analysis by Flow Cytometry

Cells were harvested and fixed with 75% alcohol overnight at −20°C, and then washed the next day with phosphate-buffered saline (PBS) prior to incubation with 0.2 mg/ml RNase (Sigma, St. Louis, MO) and 10 µg/ml propidium iodide at 37°C for 30 min. At least 15,000 stained cells were analyzed and the percentage of cells in G0/G1, S, and G2/M phases was determined by a FACS Calibur flow cytometer (Becton-Dickinson, Franklin, NJ). Experiments were performed in triplicate.

### Colony Formation Assays

Six-well plates with underlayers consisting of 0.8% agar medium were prepared. At 24 h post-transfection, cells were plated at a density of 1,000 cells per well in plates and allowed to grow for 2 weeks prior to fixation in ice-cold methanol and Giemsa’s stain. The survival fraction was determined by dividing the number of shLIN28 colonies by the number of control cell colonies. Colonies were counted under a light microscope. Experiments were performed in triplicate.

### Cell Proliferation Assays

U251 cells and U373 cells transfected with LIN28 shRNA or a negative control lentivirus were plated at a density of 1,000 cells per well in collagen-coated 96-well plates. The Cell Counting Kit-8 (CCK-8; Dojindo, Kumamoto, Japan) was used to perform cell proliferation assays. Briefly, 10 µl CCK-8 solution was added to each well and incubated at 37°C for 2 h. Optical density (OD) was then measured at 450 nm. Experiments were performed in triplicate.

### Cell Apoptosis Assays

The apoptotic response was measured using the flow cytometric method. Treated and untreated cells were harvested, washed once with PBS, and then fixed in 70% ethanol. Ethanol-fixed cells were stained with propidium iodide (16.5 mg/ml) in PBS after RNase (0.03 mg/ml) digestion. Samples were analyzed with a FACS Calibur (Becton-Dickinson, Franklin, NJ). For each sample, 10,000 cells were analyzed. Experiments were performed in triplicate.

### Statistical Analyses

For each patient, median overall survival (OS) and progression-free survival (PFS) were recorded. PFS was defined as the time from initial surgical diagnosis to tumor progression based on magnetic resonance imaging (MRI) or death from glioma. OS was defined as the time from the initial surgical diagnosis to death. PFS and OS curves were calculated using the Kaplan-Meier method and compared using the log-rank test. Univariate and multivariate analyses were performed by a stepwise backward Cox regression model. Factors with a result of p<0.2 in the univariate analysis were added into the multivariate analysis.

Data from the experiments on cultured cells were expressed as mean ± standard deviation (S.D.) and statistical significance between the groups was analyzed with one-way analysis of variance (ANOVA), followed by Dunnett’s t-test for post-hoc pair-wise comparisons. The SPSS 16.0 software (SPSS Inc., Chicago, USA) was used for the statistical analysis and the level of significance was set as p<0.05.

## Results

### Gene Microarray Analysis

**Figure 1:**
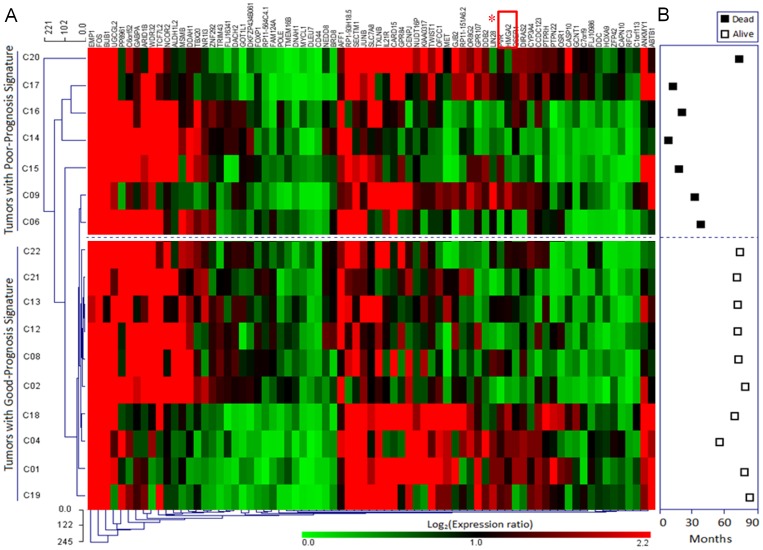
Unsupervised two-dimensional cluster analysis of 17 gliomas. A. Two-dimensional presentation of transcript ratios for 17 glioma samples. The cutoffs of fold changes ≥2.5 and Student’s t-test p≤0.011 were used to select the genes with significantly different expression. There were 75 significant genes identified across the group. Each row represents a tumor and each column a single gene. As shown in the color bar, red indicates a high level of expression of mRNA in the tumor, compared to the reference level of mRNA, and green indicates a low level of expression. The asterisk represents the gene *LIN28*. The dotted line is the previously determined threshold between a good-prognosis signature and a poor-prognosis signature. B. The total duration of follow-up for all patients.

Glioma tissue samples from 17 patients were cultured and the cRNAs were subsequently prepared and hybridized onto the Affymetrix U133 plus 2.0 array. Of the 54675 genes represented on the array, 75 genes displayed at least a 2.5-fold increase or decrease in expression at the *p*<0.011 level with a false discovery rate. Of these, 43 genes were upregulated, while 32 genes were down-regulated in tumors from patients in the poor prognosis group compared to patients from the patient group with a good prognosis (Fig. 1). The criteria used to define poor versus good prognosis gliomas included the survival status at 96 months post-procedure.

### Gene Ontology (GO) Analysis

To elucidate the relationship between differential gene expression patterns, we examined the functional bias of the 75 differentially expressed transcripts according to GO classifications. These differentially expressed transcripts were grouped into 28 GO based on biological process GO terms. The 10 most enriched GO terms (gene count >10) included organ development, system development, multicellular organismal process, anatomical structure development, immune system process, blood vessel morphogenesis, anatomical structure formation involved in morphogenesis, immune response, blood vessel development, and response to metal ions (Table S1). Analyses of GO also indicated that there were 6 GO terms identified by cellular component classification, including the extracellular region part, the extracellular space, extracellular matrix (ECM), MHC class II protein complex, intracellular part and intracellular organelle part (Table S2), as well as 5 GO terms identified by molecular function classification, including receptor binding, signaling molecule, other transcription factor, MHC class II receptor activity, and extracellular matrix binding (Table S3).

### High LIN28 Expression Correlates with Poorer Survival of Glioma Patients

**Figure 2:**
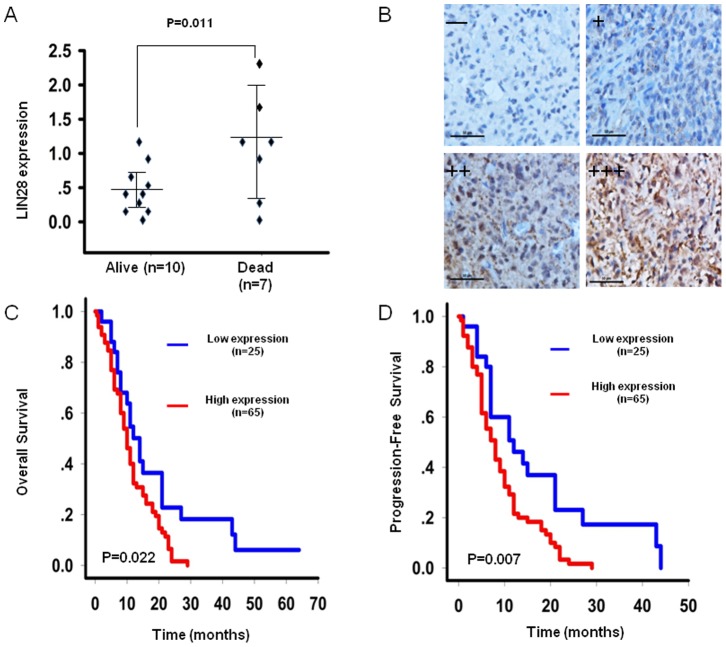
The expression of LIN28 and its correlation with overall survival and progression-free survival of glioma patients. A. The expression difference of LIN28 between the good prognosis group and poor prognosis group by gene microarray analysis. B. Immunohistochemical staining of LIN28 in GBM tissues. C,D. The Kaplan-Meier survival curve for overall survival and progression-free survival for 90 GBM patients stratified by LIN28 expression.

Our results showed that the expression levels of LIN28 in the group of patients with a poorer prognosis were significantly higher than those in the group of patients with a good prognosis by gene microarray analysis (Median LIN28 expression: 1.23 vs. 0.38, respectively; p = 0.011; Fig. 2A, Table S4).

To confirm the results obtained from our microarray analysis, we used a collection of tissues from 90 GBMs and evaluated LIN28 expression by means of immunohistochemistry (Fig. 2B). Similar findings were observed in GBM patients stratified by LIN28 expression, indicating that patients expressing high LIN28 levels had a significantly shorter OS and PFS relative to patients expressing low LIN28 levels (median OS: 10 vs. 14 months, p = 0.015; and median PFS: 8 vs. 12 months, respectively; p = 0.004; Fig. 2C,D). Moreover, the univariate analysis indicated that a high expression of LIN28 was a risk factor for a shorter OS and PFS (HR = 1.845, p = 0.022; and HR = 2.052, p = 0.007, respectively; Table 3). Furthermore, the Cox regression analysis revealed that high LIN28 expression was an independent prognostic factor for both OS and PFS in GBM patients (median OS: 10 vs. 14 months, p = 0.024; and median PFS 8 vs. 12 months, p = 0.036, respectively; Table 4).

**Table 3:** Univariate analysis of factors associated with survival and progression(n = 90).

Variable	GBM					
	OS			PFS		
	HR	95% CI	P	HR	95% CI	P
Age (>50 vs. ≤50 y)	1.869	1.166–2.997	0.009	1.700	1.065–2.713	0.026
Lin28 (High vs. low)	1.845	1.092–3.117	0.022	2.052	1.215–3.466	0.007
IICP (Yes vs. no)	0.929	0.605–1.428	0.738	0.869	0.562–1.345	0.530
MTD (<5 vs ≥5 cm)	0.645	0.426–0.977	0.039	0.804	0.538–1.202	0.287
Cystic Degeneration(Yes vs. no)	0.683	0.448–1.041	0.076	0.762	0.489–1.187	0.229
Necrosis (Yes vs. no)	0.956	0.525–1.738	0.882	1.139	0.629–2.064	0.668
Chemotherapy(Yes vs. no)	0.727	0.457–1.156	0.178	0.837	0.523–1.341	0.460
Radiotherapy(Yes vs. no)	0.551	0.346–0.878	0.012	0.546	0.341–0.875	0.012
Resection Degree	NA	NA	0.029	NA	NA	0.047
Subtotal vs. Total	0.713	0.420–1.209	0.210	0.645	0.379–1.099	0.107
Partial vs. Total	3.837	1.178–12.496	0.026	2.824	0.874–9.122	0.083
Seizure (Yes vs. no)	0.820	0.407–1.651	0.578	1.064	0.531–2.131	0.862
Gender(Female vs. male)	0.821	0.519–1.298	0.398	0.719	0.448–1.154	0.171

Abbreviations: IICP, increased intracranial pressure; MTD, mean tumor diameter; NA, not applicable; HR, Hazard ratio; OS, overall survival; PFS, progression-free survival.

**Table 4:** Multivariate analysis of factors associated with survival and progression (n = 90).

Survival*	Median Survival (months, 95% CI)		HR	95% CI	*P*
OS					
Age (>50 vs. ≤50 y)	10(8.357–11.643)	15(8.845–21.155)	2.053	1.267–3.326	0.003
Lin28 (High vs. low)	10(8.182–11.818)	14(10.467–17.533)	1.874	1.085–3.237	0.024
Resection	NA	NA	NA	NA	0.018
Subtotal vs. Total	13(8.734–17.266)	10(8.271–11.729)	0.651	0.382–1.109	0.114
Partial vs. Total	2(0.400–3.600)	10(8.271–11.729)	3.878	1.164–12.918	0.027
Radiotherapy (Yes vs. No)	12(10.360–13.640)	9(5.998–12.002)	0.541	0.336–0.873	0.012
MTD (<5 vs ≥5 cm)	10(7.816–12.184)	11(9.431–12.569)	0.575	0.369–0.895	0.014
PFS					
Age (>50 vs. ≤50 y)	7(5.135–8.865)	10(6.183–13.817)	1.865	1.151–3.022	0.011
Lin28 (High vs. low)	8(6.044–9.956)	12(4.350–19.650)	1.798	1.040–3.110	0.036
Resection	NA	NA	NA	NA	0.036
Subtotal vs. Total	12(7.734–16.266)	7(5.531–8.469)	0.546	0.316–0.944	0.030
Partial vs. Total	1(NA)	7(5.531–8.469)	2.060	0.625–6.785	0.235
Radiotherapy (Yes vs. No)	10(6.736–13.264)	7(3.183–10.817)	0.445	0.268–0.739	0.002
Gender(Female vs. male)	9(5.883–12.117)	7(4.449–9.551)	0.589	0.353–0.984	0.043

Abbreviations: IICP, increased intracranial pressure; MTD, mean tumor diameter; NA, not applicable.

* Variables were adopted for their prognostic significance by univariate analysis (P≤0.2).

### Lentivirus-based RNAi of LIN28 Down-regulates LIN28 Expression

**Figure 3:**
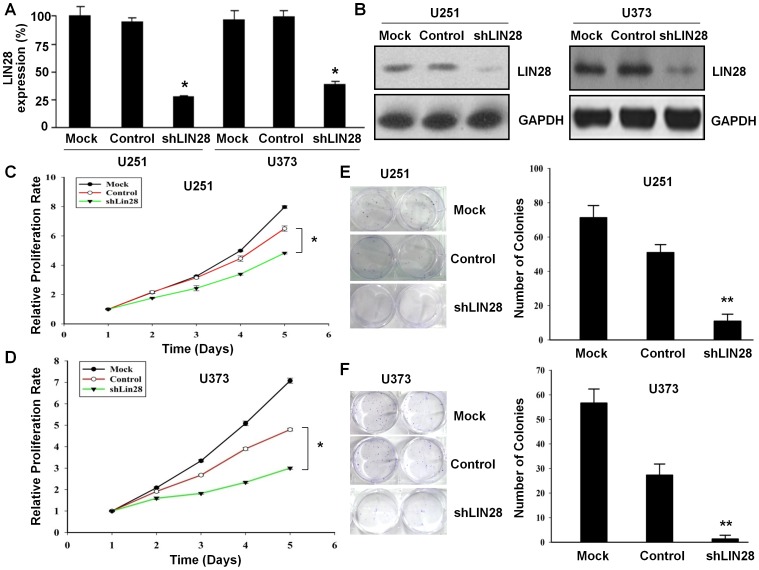
Decreased expression of LIN28 mRNA and protein in human glioma cells by siRNA. U251 and U373 cells were transfected with lentivirus-based small interfering RNA (shLIN28) or the empty plasmid (as the negative control, Control). A,B. RT-PCR and Western blot analysis were used to detect the LIN28 mRNA and protein expression levels in U251 and U373 cells. C,D. CCK-8 assays were used to determine the effects of LIN28 knockdown on cell proliferation in U251 and U373 cells. E,F. The effects of LIN28 knockdown on cell colony formation in U251 and U373 cells. * *p*<0.05, ** *p*<0.01.

To investigate the role of LIN28 in the growth of human glioma cells, we generated LIN28-knockdown stable cell lines as well as the corresponding control and normal glioma cell lines (designated as shLIN28, control, and MOCK, respectively). As shown in Figures 3A and B, both LIN28 mRNA and protein expression were significantly reduced by RNAi-mediated LIN28 knockdown in U251 and U373 cell lines (*p*<0.05).

### RNAi of LIN28 Suppresses Glioma Cell Growth and Colony Formation *in vitro*

To assess the role of LIN28 in the growth of human glioma cells, we performed MTT assays to examine the cell proliferation of U251 cells and U373 cells after transfection with LIN28 shRNA. As shown in Figures 3C and D, down-regulation of LIN28 by RNAi caused a significant delay of both U251 and U373 cell growth after 3 days post-transfection compared to the negative control (*p*<0.05).

To determine the effects of LIN28 in human glioma tumorigenesis *in vitro*, we assayed the colony formation of U251 and U373 cells in soft agar. As shown in Figures 3E and F, reduction of LIN28 in U251 and U373 cells lines remarkably reduced the number of colonies from 27.3±4.5 to 1.3±1.5 (*p*<0.01) and 51.0±4.6 to 11.0±4.0 (*p*<0.01) compared to control, respectively. These results indicated that LIN28 might be involved in human glioma cell growth and tumorigenesis *in vitro*.

### LIN28 Regulates the Cell Cycle and Induces Apoptosis in Glioma Cells

**Figure 4:**
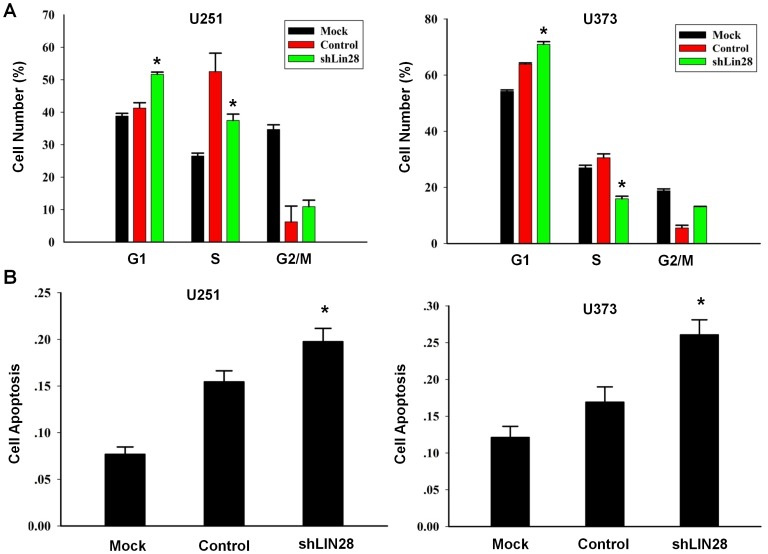
Cell cycle distribution and apoptosis of human glioma cells with shRNA-mediated knockdown of LIN28. A. Suppression of LIN28 leads to the cell cycle arrest of U251 and U373 cells treated with LIN28 shRNA in the G1 phase, accompanied by a decreased population in S phase. B. Relative to the controls, the percentage of apoptotic cells was significantly increased in U251 cells treated with LIN28 shRNA. The changes were also significant in U373 cells. * *p*<0.05.

LIN28 has been shown to regulate the translation of cell cycle proteins in other cells types [27]. The cell cycle distribution is highly indicative of changes in cell proliferation, apoptosis, differentiation, and senescence, which may lead to carcinogenesis. Thus, we assess LIN28-mediated changes in the cell cycle. As shown in Figure 4A, suppression of LIN28 with shRNA led to a modest but consistent increase in the population of U251 cells in G1 phase compared to control (51.67±0.70% vs. 41.62±1.67%, respectively). This was accompanied by a decreased percentage of cells in S phase compared to control (37.45±1.97% vs. 52.52±5.66%, respectively). In U373 cells, suppression of LIN28 also caused an increase in the percentage of cells in G1 phase (70.95±0.95% vs. 63.93±0.41%) and a decreased percentage in S phase (15.92±0.92% vs. 30.54±1.39%) compared to control, respectively. These results indicate that LIN28 may play an active role in glioma cell cycle progression, which, in turn, accelerates cell proliferation.

To determine the role of LIN28 in apoptosis of human glioma cells, we performed apoptosis assays using a flow cytometric method. As shown in Figure 4B, apoptotic cells were significantly increased from 15.45±1.18% to 19.76±1.42% in U251 cells (*p*<0.05) and from 16.92±2.07% to 26.07±2.04% in U373 cells (*p*<0.05) after transfection with LIN28 shRNA relative to control, respectively. These results indicate that LIN28 might be involved in human glioma cell apoptosis.

## Discussion

LIN28, a highly conserved RNA-binding protein, was initially described as a regulator of developmental timing sequence in *Caenorhabditis elegans* [30]. The mammalian homolog of LIN28 is also implicated in developmental processes. It is highly expressed in embryonic stem cells and down-regulated during differentiation [31]. LIN28 consists of a cold shock domain (CSD) and a pair of retroviral-type CCHC zinc fingers [30], [31], which bind to the terminal loops of the precursors of let-7 family miRNAs and block their processing into mature miRNAs [6]–[8]. Let-7 is known to regulate several oncogenes, including Ras [9], c-Myc [10], and Hmga2 [11], and the repression of let-7 has been linked to several tumor types, such as lung [9], [10], breast [12], and ovarian cancer [13]–[15]. Thus, let-7 may be involved in tumor inhibition. In addition, LIN28 has been shown to be involved in the post-transcriptional regulation of the let-7 miRNA family, and therefore LIN28 is likely to be involved in tumor occurrence and development [16].

When expressed at an appropriate level, LIN28 was found to reprogram human somatic fibroblast to pluripotency together with OCT4, SOX2, and NANOG, while the overexpression of LIN28 induced malignant transformation of cells [16]. A previous study found high expression of LIN28 in approximately 15% of more than 500 primary tumors or cell lines analyzed [16], especially in the subset of tumors that were poorly differentiated and had a worse prognosis, such as blast phase chronic myeloid leukemia [16], hepatocellular carcinoma (HCC) [16]–[18], ovarian carcinoma [13]–[15], colon carcinoma [19], [20], and germ cell carcinoma [21]–[23]. Furthermore, overexpression of LIN28 is associated with high serum a-fetoprotein (AFP) levels and high-grade HCC tumors [17] as well as a reduced overall survival and increased probability of tumor recurrence in colon carcinoma [19].

In the present study, we examined the expression of LIN28 by gene microarray analysis and immunohistochemistry of a tissue microarray and found that high expression of LIN28 was associated with reduced OS and PFS. Univariate and multivariate analyses showed that LIN28 acts as an independent prognostic factor for GBM patients. The *in vitro* experiments indicated that LIN28 can increase U251 and U373 cell growth and enhance the colony formation ability. Furthermore, LIN28 promotes cell cycle progression from G1 to S phase and increases the percentage of cells in G2/M. Moreover, LIN28 inhibits glioma cell apoptosis. Taken together, these results indicate that LIN28 may be an oncogene and promotes glioma cell growth mainly through the promotion of cell cycle progression and inhibition of cell apoptosis.

Dysfunction of cell cycle and apoptosis regulation plays an important role in the development of a tumor, including glioma. In the three main signaling pathways defined by the TCGA group, the RB and p53 pathway are critical for regulating the cell cycle, while the p53 pathway is also associated with cell apoptosis. It has been shown that LIN28 can modulate the transcription of CDK4 in the RB pathway, and more recently, bioinformatics analysis found that p53 may increase the expression of let-7, which could suppress the expression of LIN28 [32]. However, the precise molecular mechanisms involved in the p53 and LIN28 interaction in glioma are currently unknown and will require further experimentation.

Many studies have focused on targeted molecular therapies, but the results are disappointing with relatively rare radiographic responses and no significant prolongation of PFS reported in GBM [33], [34]. This is partially due to the coactivation of several signaling pathways during glioma development. Therefore, identification of new targets, especially those involved in the three core pathways responsible for GBM development, is of utmost importance. Because LIN28 has been shown to be involved in each of these three pathways, the development of therapeutics targeting this factor may provide improved efficacy for treating GBM. In addition, LIN28 is a stem cell reprogramming factor, and thus agents targeting LIN28 may also target glioma stem cells.

In conclusion, we have demonstrated that high expression of LIN28 in glioma is associated with a shorter median OS and PFS and that LIN28 might be a predictor of prognosis in GBM patients, thus serving as an independent prognostic factor. In addition, suppression of LIN28 reduced the growth of glioma cells and promoted apoptosis. Its involvement in the three core signaling pathways involved in GBM makes it a potential target for cancer therapy as an approach to overcome the poor options currently available for these patients.

## Supporting Information

Table S1 **10 Gene Ontology based on biological process Gene Ontology terms.**
(DOC)

Table S2 **6 Gene Ontology terms identified by cellular component classification.**
(DOC)

Table S3 **5 Gene Ontology terms identified by molecular function classification.**
(DOC)

Table S4 **The expression levels of 75 differentially expressed genes in good and poor prognosis gliomas.**
(DOC)
