# Supplementary material for: LIN28 Is Involved in Glioma Carcinogenesis and Predicts Outcomes of Glioblastoma Multiforme Patients
Source: PLoS One. 2014 Jan 24;9(1):e86446. doi: 10.1371/journal.pone.0086446 (PMC3901701; doi:10.1371/journal.pone.0086446)
Supplement: Table S1 — 10 Gene Ontology based on biological process Gene Ontology terms. (DOC) [file pone.0086446.s001.doc]

| **Table S1.** **10 Gene Ontology based on biological process Gene Ontology terms.** | | | | | |
| --- | --- | --- | --- | --- | --- |
| **Biological process** | **Genes** | **Count** | **%** | **P-Value** | **Benjamini** |
| organ development | 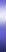 | 24 | 43.6 | 2.80E-03 | 5.40E-01 |
| system development | 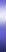 | 18 | 32.7 | 1.30E-02 | 5.50E-01 |
| multicellular organismal process | 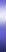 | 17 | 30.9 | 1.50E-03 | 4.70E-01 |
| anatomical structure development | 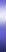 | 17 | 30.9 | 3.60E-03 | 5.30E-01 |
| immune system process | 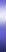 | 17 | 30.9 | 1.30E-02 | 5.50E-01 |
| blood vessel morphogenesis | 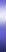 | 15 | 27.3 | 7.10E-04 | 4.50E-01 |
| anatomical structure formation involved in morphogenesis | 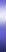 | 11 | 20 | 3.10E-02 | 7.40E-01 |
| immune response | 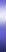 | 11 | 20 | 3.90E-02 | 7.80E-01 |
| blood vessel development | 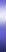 | 10 | 18.2 | 3.70E-03 | 4.60E-01 |
| response to metal ion | 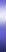 | 10 | 16.4 | 3.40E-02 | 7.60E-01 |
