# Supplementary material for: LIN28 Is Involved in Glioma Carcinogenesis and Predicts Outcomes of Glioblastoma Multiforme Patients
Source: PLoS One. 2014 Jan 24;9(1):e86446. doi: 10.1371/journal.pone.0086446 (PMC3901701; doi:10.1371/journal.pone.0086446)
Supplement: Table S2 — 6 Gene Ontology terms identified by cellular component classification. (DOC) [file pone.0086446.s002.doc]

**Table S2. 6 Gene Ontology terms identified by cellular component classification.**

| **Term** | **Genes** | **Count** | **%** | **P-Value** | Benjamini |
| --- | --- | --- | --- | --- | --- |
| | extracellular region part | | --- | | 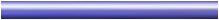 | 38 | 69.1 | 9.90E-02 | 9.20E-01 |
| | 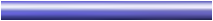extracellular space | | --- | |  | 37 | 67.3 | 9.90E-02 | 9.50E-01 |
| | extracellular matrix | | --- | | 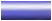 | 8 | 14.5 | 2.20E-02 | 9.60E-01 |
| | MHC class II protein complex | | --- | | 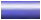 | 6 | 10.9 | 5.10E-02 | 9.80E-01 |
| | intracellular part | | --- | | 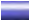 | 4 | 7.3 | 8.20E-02 | 9.80E-01 |
| | intracellular | | --- | | 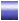 | 2 | 3.6 | 8.20E-02 | 9.50E-01 |
