# Supplementary material for: LIN28 Is Involved in Glioma Carcinogenesis and Predicts Outcomes of Glioblastoma Multiforme Patients
Source: PLoS One. 2014 Jan 24;9(1):e86446. doi: 10.1371/journal.pone.0086446 (PMC3901701; doi:10.1371/journal.pone.0086446)
Supplement: Table S3 — 5 Gene Ontology terms identified by molecular function classification. (DOC) [file pone.0086446.s003.doc]

| **Table S3. 5 Gene Ontology terms identified by molecular function classification.** | | | | | |
| --- | --- | --- | --- | --- | --- |
| **Term** | **Genes** | **Count** | **%** | **P-Value** | **Benjamini** |
| receptor binding | 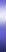 | 7 | 12.7 | 5.50E-02 | 1.00E+00 |
| signaling molecule | 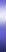 | 6 | 10.9 | 9.40E-02 | 1.00E+00 |
| other transcription factor | 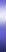 | 4 | 7.3 | 9.80E-02 | 9.60E-01 |
| MHC class II receptor activity | 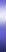 | 2 | 3.6 | 5.70E-02 | 1.00E+00 |
| extracellular matrix binding | 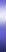 | 2 | 3.6 | 8.10E-02 | 1.00E+00 |
