# Supplementary material for: LIN28 Is Involved in Glioma Carcinogenesis and Predicts Outcomes of Glioblastoma Multiforme Patients
Source: PLoS One. 2014 Jan 24;9(1):e86446. doi: 10.1371/journal.pone.0086446 (PMC3901701; doi:10.1371/journal.pone.0086446)
Supplement: Table S4 — The expression levels of 75 differentially expressed genes in good and poor prognosis gliomas. (DOC) [file pone.0086446.s004.doc]

**Table S4. The expression levels of 75 differentially expressed genes in good and poor prognosis gliomas.**

| **Gene**  **Symbole** | **Good-prognosis gliomas** | | | | | | | | | | **Poor-prognosis gliomas** | | | | | | |
| --- | --- | --- | --- | --- | --- | --- | --- | --- | --- | --- | --- | --- | --- | --- | --- | --- | --- |
|  | C01 | C02 | C04 | C08 | C12 | C13 | C18 | C19 | C21 | C22 | C06 | C09 | C14 | C15 | C16 | C17 | C20 |
| TRIM43 | 1.15 | 0.87 | 1.00 | 0.14 | 0.57 | 0.62 | 0.19 | 0.54 | 1.15 | 0.93 | 0.20 | 0.41 | 0.10 | 0.18 | 0.15 | 0.31 | 0.25 |
| GOT1L1 | 0.50 | 0.76 | 0.06 | 0.87 | 0.33 | 0.62 | 0.38 | 0.66 | 0.93 | 0.93 | 0.71 | 0.06 | 0.05 | 0.09 | 0.29 | 0.09 | 0.04 |
| CCDC123 | 0.11 | 0.76 | 1.07 | 0.93 | 0.14 | 0.16 | 0.62 | 0.07 | 0.09 | 1.07 | 0.93 | 0.62 | 1.32 | 1.23 | 1.41 | 1.32 | 1.15 |
| ALDH1L2 | 2.46 | 2.83 | 2.83 | 0.47 | 2.46 | 2.14 | 0.44 | 4.59 | 0.66 | 4.59 | 0.44 | 0.50 | 0.44 | 0.33 | 0.38 | 0.44 | 0.50 |
| ARID1B | 5.66 | 6.50 | 2.83 | 3.03 | 4.00 | 3.03 | 8.57 | 1.62 | 1.07 | 3.73 | 1.23 | 2.64 | 1.23 | 0.41 | 0.87 | 0.44 | 0.50 |
| POLE | 0.38 | 0.54 | 0.41 | 0.44 | 0.09 | 0.50 | 0.33 | 0.29 | 0.19 | 0.76 | 0.09 | 0.09 | 0.08 | 0.06 | 0.31 | 0.10 | 0.09 |
| PP8961 | 0.47 | 5.28 | 0.50 | 7.46 | 8.00 | 4.00 | 3.25 | 2.83 | 6.06 | 4.00 | 0.19 | 0.44 | 1.87 | 2.83 | 0.50 | 0.41 | 0.44 |
| OFCC1 | 0.54 | 1.07 | 0.29 | 0.27 | 1.32 | 0.31 | 0.47 | 0.31 | 0.50 | 0.54 | 1.32 | 0.54 | 1.23 | 1.87 | 2.00 | 3.03 | 2.30 |
| TBX20 | 0.87 | 1.32 | 0.35 | 1.23 | 1.41 | 1.87 | 1.15 | 1.52 | 0.66 | 2.00 | 0.22 | 0.31 | 0.54 | 0.13 | 0.23 | 0.54 | 0.87 |
| DLEU7 | 0.19 | 0.23 | 0.16 | 0.38 | 0.16 | 0.05 | 0.16 | 0.23 | 0.12 | 0.23 | 0.05 | 0.07 | 0.05 | 0.02 | 0.03 | 0.08 | 0.07 |
| AFF1 | 0.66 | 3.48 | 2.64 | 1.32 | 3.03 | 2.00 | 2.46 | 1.41 | 2.64 | 2.30 | 1.07 | 0.87 | 0.71 | 1.87 | 0.87 | 0.81 | 1.15 |
| RP11-151A6.2 | 0.27 | 0.66 | 0.38 | 0.31 | 1.52 | 0.54 | 0.33 | 0.25 | 0.31 | 0.23 | 0.93 | 1.41 | 0.76 | 1.32 | 0.20 | 2.00 | 1.52 |
| OR8G2 | 0.44 | 0.38 | 0.50 | 0.29 | 1.41 | 0.47 | 0.33 | 1.00 | 0.35 | 0.41 | 1.74 | 2.64 | 0.44 | 1.74 | 0.41 | 1.41 | 1.23 |
| DNAH1 | 0.38 | 0.50 | 0.44 | 0.11 | 0.29 | 0.33 | 0.41 | 0.29 | 0.54 | 0.08 | 0.20 | 0.04 | 0.03 | 0.05 | 0.18 | 0.31 | 0.05 |
| HMGA2 | 0.57 | 0.20 | 0.27 | 0.81 | 0.41 | 0.13 | 0.57 | 0.29 | 0.71 | 0.50 | 1.62 | 0.54 | 1.15 | 1.23 | 0.71 | 1.52 | 0.31 |
| NUDT16P | 0.41 | 0.41 | 0.44 | 0.38 | 0.41 | 0.35 | 0.71 | 0.57 | 0.57 | 0.50 | 1.00 | 0.50 | 0.33 | 2.64 | 2.14 | 1.41 | 1.52 |
| FOXP1 | 0.66 | 0.62 | 0.81 | 0.31 | 0.76 | 0.23 | 0.54 | 0.54 | 0.35 | 0.50 | 0.20 | 0.50 | 0.22 | 0.18 | 0.08 | 0.16 | 0.23 |
| NR1I3 | 0.25 | 1.62 | 0.41 | 0.87 | 1.52 | 1.07 | 1.41 | 1.32 | 1.74 | 0.93 | 0.31 | 0.47 | 0.20 | 0.38 | 0.27 | 0.31 | 0.47 |
| EMP1 | 13.00 | 2.00 | 9.85 | 9.85 | 4.59 | 16.00 | 4.92 | 32.00 | 1.62 | 24.25 | 4.92 | 19.70 | 45.25 | 48.50 | 51.98 | 84.45 | 17.15 |
| JUNB | 1.15 | 1.32 | 0.81 | 1.32 | 1.00 | 1.07 | 2.83 | 2.00 | 2.64 | 0.76 | 0.87 | 1.52 | 3.73 | 4.59 | 9.19 | 9.85 | 5.66 |
| MET | 0.05 | 0.19 | 0.05 | 0.07 | 0.66 | 0.06 | 0.66 | 0.04 | 1.52 | 0.09 | 1.15 | 0.41 | 1.23 | 1.62 | 1.62 | 3.03 | 0.57 |
| GCNT1 | 0.29 | 0.13 | 0.13 | 0.12 | 0.33 | 0.10 | 0.06 | 0.11 | 0.16 | 0.35 | 0.13 | 0.54 | 0.50 | 0.23 | 0.47 | 1.32 | 0.76 |
| CYP3A4 | 0.27 | 0.22 | 0.31 | 1.15 | 0.47 | 0.22 | 1.07 | 0.47 | 0.33 | 0.23 | 1.15 | 1.00 | 1.15 | 1.23 | 0.71 | 0.35 | 1.23 |
| PTPRH | 0.35 | 0.38 | 0.35 | 0.87 | 0.23 | 0.41 | 0.50 | 0.38 | 0.87 | 0.35 | 0.66 | 1.41 | 1.00 | 0.57 | 1.07 | 2.46 | 1.00 |
| FOS | 11.31 | 3.03 | 5.66 | 8.57 | 2.64 | 6.06 | 6.50 | 9.85 | 17.15 | 7.46 | 3.48 | 4.92 | 11.31 | 36.76 | 36.76 | 34.30 | 32.00 |
| BUB1 | 2.00 | 6.06 | 3.48 | 14.93 | 14.93 | 9.19 | 16.00 | 3.73 | 0.66 | 6.06 | 10.56 | 11.31 | 27.86 | 16.00 | 34.30 | 18.38 | 9.85 |
| MSMB | 3.03 | 2.14 | 2.00 | 1.62 | 0.81 | 1.32 | 3.73 | 0.33 | 1.87 | 0.71 | 0.38 | 0.66 | 0.25 | 0.35 | 0.62 | 0.71 | 0.44 |
| BRD8 | 0.10 | 0.44 | 1.07 | 0.66 | 0.57 | 0.76 | 0.62 | 0.44 | 0.62 | 0.44 | 0.38 | 0.07 | 0.25 | 0.25 | 0.18 | 0.07 | 0.10 |
| CASP10 | 0.23 | 0.16 | 0.25 | 0.22 | 0.13 | 0.22 | 0.16 | 0.20 | 0.47 | 0.23 | 0.62 | 1.32 | 0.16 | 0.20 | 1.07 | 0.81 | 2.30 |
| SECTM1 | 2.14 | 0.50 | 1.87 | 2.83 | 1.00 | 0.50 | 2.83 | 1.74 | 0.71 | 1.00 | 4.00 | 2.14 | 7.46 | 4.92 | 8.57 | 10.56 | 5.66 |
| TWIST1 | 0.27 | 0.76 | 0.81 | 0.66 | 0.93 | 0.33 | 0.47 | 0.31 | 0.81 | 0.81 | 0.87 | 1.87 | 2.30 | 1.07 | 2.83 | 3.25 | 1.41 |
| HOXA9 | 0.31 | 0.03 | 0.07 | 0.07 | 0.19 | 0.07 | 0.09 | 0.19 | 0.38 | 0.13 | 0.22 | 0.50 | 0.44 | 0.47 | 0.22 | 0.38 | 0.27 |
| NCOR2 | 0.54 | 6.50 | 4.00 | 2.30 | 2.64 | 5.66 | 0.87 | 3.48 | 0.71 | 5.28 | 0.71 | 0.76 | 0.44 | 0.62 | 0.66 | 0.66 | 1.00 |
| MYCL1 | 0.31 | 0.16 | 0.20 | 0.31 | 0.25 | 0.07 | 0.20 | 0.27 | 0.41 | 0.41 | 0.05 | 0.04 | 0.06 | 0.18 | 0.03 | 0.11 | 0.13 |
| PVR | 0.93 | 0.06 | 0.10 | 0.35 | 0.13 | 0.31 | 0.12 | 1.07 | 0.66 | 0.14 | 1.07 | 0.71 | 1.00 | 0.81 | 1.23 | 1.00 | 0.87 |
| SLC7A8 | 0.57 | 0.57 | 0.66 | 0.62 | 0.57 | 0.54 | 0.47 | 1.62 | 2.30 | 3.03 | 5.66 | 6.96 | 3.73 | 0.54 | 1.52 | 1.74 | 4.00 |
| LIN28 | 1.07 | 0.18 | 0.18 | 0.71 | 0.07 | 0.35 | 0.62 | 0.41 | 0.62 | 0.25 | 2.30 | 1.00 | 1.62 | 1.23 | 1.23 | 0.44 | 0.27 |
| CARD15 | 2.00 | 0.27 | 0.44 | 0.50 | 0.47 | 0.50 | 3.48 | 0.50 | 0.54 | 0.71 | 6.50 | 2.30 | 2.30 | 0.41 | 2.83 | 4.92 | 6.06 |
| TMEM16B | 0.25 | 0.57 | 0.50 | 0.29 | 0.19 | 0.25 | 0.38 | 0.44 | 0.35 | 0.31 | 0.07 | 0.08 | 0.08 | 0.27 | 0.41 | 0.20 | 0.04 |
| C1orf113 | 0.10 | 0.14 | 0.31 | 0.10 | 0.16 | 0.09 | 0.11 | 0.13 | 0.22 | 0.13 | 0.71 | 0.25 | 0.33 | 0.19 | 0.44 | 0.57 | 0.47 |
| GPR107 | 0.22 | 0.87 | 1.41 | 0.27 | 0.14 | 0.14 | 0.71 | 1.00 | 0.16 | 0.06 | 1.32 | 1.07 | 2.14 | 1.15 | 1.87 | 0.81 | 1.74 |
| CAPN10 | 0.20 | 0.11 | 0.13 | 0.12 | 0.15 | 0.27 | 0.03 | 0.05 | 0.06 | 0.16 | 0.11 | 0.71 | 0.44 | 0.62 | 0.38 | 0.14 | 0.57 |
| C7orf9 | 0.10 | 0.10 | 0.41 | 0.12 | 0.11 | 0.15 | 0.05 | 0.20 | 0.18 | 0.12 | 0.50 | 0.93 | 0.71 | 0.12 | 0.09 | 0.54 | 0.62 |
| IL21R | 0.38 | 0.57 | 0.66 | 2.83 | 0.41 | 0.47 | 1.41 | 0.35 | 0.76 | 0.66 | 4.92 | 0.54 | 4.00 | 0.35 | 3.48 | 2.00 | 4.92 |
| GJB2 | 0.50 | 0.71 | 1.32 | 0.09 | 0.66 | 0.13 | 0.05 | 0.06 | 0.66 | 0.20 | 1.32 | 1.41 | 0.44 | 0.38 | 2.64 | 2.30 | 2.30 |
| GPR84 | 0.29 | 0.47 | 1.41 | 2.00 | 0.87 | 0.22 | 0.38 | 1.23 | 0.41 | 1.00 | 3.25 | 2.46 | 0.54 | 0.33 | 5.28 | 4.92 | 2.30 |
| ABTB1 | 0.47 | 0.57 | 0.57 | 0.35 | 0.44 | 0.41 | 0.47 | 2.46 | 0.93 | 0.62 | 3.48 | 2.30 | 2.14 | 1.52 | 2.46 | 2.46 | 0.81 |
| TXLNB | 2.64 | 0.87 | 1.41 | 2.30 | 0.31 | 0.76 | 0.38 | 1.23 | 2.00 | 3.48 | 4.29 | 0.38 | 4.92 | 1.87 | 5.28 | 6.96 | 3.25 |
| OSR1 | 0.10 | 0.15 | 0.81 | 0.09 | 0.08 | 0.08 | 0.76 | 0.06 | 0.18 | 0.13 | 0.44 | 0.62 | 0.62 | 0.31 | 1.23 | 1.62 | 0.76 |
| RP1-93H18.5 | 1.74 | 0.25 | 0.81 | 2.30 | 1.15 | 2.30 | 4.59 | 4.59 | 1.87 | 0.38 | 1.23 | 4.92 | 9.85 | 3.25 | 6.50 | 6.96 | 5.28 |
| RFC3 | 0.05 | 0.04 | 0.29 | 0.12 | 0.25 | 0.04 | 0.15 | 0.05 | 0.09 | 0.06 | 0.29 | 0.29 | 0.25 | 0.22 | 0.25 | 0.18 | 0.38 |
| ANKMY1 | 0.71 | 1.74 | 0.41 | 0.31 | 0.66 | 0.47 | 0.33 | 2.14 | 1.74 | 0.50 | 0.87 | 3.03 | 1.32 | 2.83 | 1.62 | 3.25 | 2.14 |
| FLJ16341 | 0.76 | 0.81 | 0.71 | 0.71 | 0.62 | 0.87 | 0.25 | 0.06 | 0.35 | 0.76 | 0.33 | 0.47 | 0.15 | 0.47 | 0.11 | 0.04 | 0.11 |
| TCF7L2 | 4.29 | 4.00 | 3.25 | 1.07 | 2.83 | 6.50 | 3.48 | 3.03 | 2.64 | 4.92 | 0.41 | 1.07 | 2.30 | 0.41 | 0.33 | 2.30 | 3.73 |
| UGCGL2 | 13.0 | 12.10 | 8.00 | 6.96 | 1.07 | 4.29 | 8.57 | 2.46 | 8.57 | 4.92 | 1.52 | 2.00 | 0.27 | 1.00 | 1.87 | 2.30 | 5.66 |
| CENPJ | 1.87 | 1.62 | 0.33 | 0.87 | 1.52 | 0.50 | 0.41 | 0.35 | 0.47 | 0.09 | 2.30 | 0.57 | 1.74 | 2.64 | 2.14 | 2.00 | 3.48 |
| CD44 | 0.16 | 0.35 | 0.11 | 0.11 | 0.12 | 0.14 | 0.13 | 0.27 | 0.15 | 0.20 | 0.05 | 0.04 | 0.04 | 0.06 | 0.09 | 0.10 | 0.03 |
| FLJ10986 | 0.27 | 0.18 | 0.20 | 0.66 | 0.13 | 0.13 | 0.10 | 0.27 | 0.41 | 0.15 | 0.35 | 0.71 | 0.38 | 0.57 | 0.71 | 0.22 | 0.71 |
| ZNF292 | 0.81 | 1.07 | 0.81 | 0.47 | 0.29 | 0.66 | 1.15 | 0.47 | 0.87 | 1.23 | 0.71 | 0.47 | 0.19 | 0.31 | 0.20 | 0.35 | 0.23 |
| PTPN22 | 0.33 | 0.71 | 0.62 | 0.06 | 0.07 | 0.12 | 0.54 | 0.13 | 0.08 | 0.02 | 0.20 | 0.66 | 0.71 | 0.54 | 2.30 | 1.74 | 1.07 |
| DDC | 0.20 | 0.12 | 0.12 | 0.50 | 0.41 | 0.11 | 0.11 | 0.11 | 0.13 | 0.29 | 0.31 | 0.33 | 0.47 | 0.50 | 0.47 | 0.41 | 0.38 |
| DKFZP434B061 | 0.54 | 0.81 | 0.27 | 0.76 | 0.35 | 1.23 | 0.23 | 0.71 | 0.50 | 0.44 | 0.35 | 0.14 | 0.14 | 0.18 | 0.27 | 0.05 | 0.35 |
| DDB2 | 0.50 | 0.35 | 0.22 | 0.16 | 0.23 | 0.19 | 0.19 | 1.32 | 0.29 | 0.19 | 0.93 | 1.52 | 1.15 | 2.14 | 0.41 | 0.33 | 1.15 |
| DACH2 | 1.00 | 0.71 | 0.87 | 0.87 | 0.44 | 0.87 | 0.35 | 0.08 | 0.93 | 0.93 | 0.81 | 0.33 | 0.23 | 0.13 | 0.08 | 0.11 | 0.29 |
| DIRAS2 | 0.93 | 0.33 | 0.57 | 0.81 | 0.57 | 0.16 | 0.13 | 0.38 | 0.38 | 0.81 | 1.00 | 1.52 | 0.31 | 1.41 | 1.41 | 0.87 | 1.32 |
| WDR32 | 6.50 | 3.73 | 2.64 | 4.29 | 2.46 | 4.59 | 2.83 | 2.64 | 4.00 | 4.92 | 1.74 | 3.73 | 0.15 | 1.41 | 0.25 | 0.62 | 2.00 |
| RP11-564C4.1 | 0.66 | 0.27 | 0.47 | 0.50 | 0.87 | 0.38 | 0.41 | 0.29 | 0.50 | 0.18 | 0.33 | 0.15 | 0.25 | 0.06 | 0.02 | 0.25 | 0.14 |
| FAM124A | 0.57 | 0.38 | 0.81 | 0.31 | 0.66 | 0.18 | 0.76 | 0.33 | 0.71 | 0.07 | 0.19 | 0.04 | 0.33 | 0.07 | 0.19 | 0.15 | 0.14 |
| ZFP42 | 0.19 | 0.35 | 0.15 | 0.07 | 0.05 | 0.14 | 0.09 | 0.09 | 0.14 | 0.31 | 0.06 | 0.54 | 0.41 | 0.71 | 0.31 | 0.44 | 0.33 |
| GABPA | 6.96 | 1.74 | 6.96 | 2.64 | 2.64 | 2.46 | 6.06 | 4.29 | 0.54 | 1.00 | 0.50 | 1.00 | 0.93 | 0.41 | 1.00 | 0.57 | 0.93 |
| DDAH1 | 2.14 | 2.83 | 1.41 | 1.52 | 0.57 | 2.30 | 1.62 | 2.46 | 1.32 | 0.87 | 0.38 | 0.87 | 0.33 | 0.57 | 0.20 | 0.35 | 0.87 |
| NEDD8 | 0.41 | 0.25 | 1.15 | 0.76 | 0.76 | 1.00 | 0.19 | 0.41 | 0.50 | 0.62 | 0.11 | 0.09 | 0.08 | 0.23 | 0.09 | 0.29 | 0.44 |
| C6orf52 | 2.46 | 2.00 | 2.83 | 8.57 | 3.03 | 4.92 | 8.00 | 2.46 | 5.28 | 2.30 | 1.07 | 0.62 | 1.32 | 1.23 | 0.29 | 0.47 | 1.62 |
| KIAA0317 | 1.52 | 0.41 | 0.50 | 1.23 | 0.50 | 0.54 | 0.18 | 0.31 | 1.23 | 0.41 | 0.87 | 1.07 | 0.23 | 2.30 | 2.30 | 2.30 | 2.64 |
| QSER1 | 0.22 | 0.18 | 0.29 | 0.76 | 0.23 | 0.62 | 0.14 | 0.22 | 0.54 | 1.07 | 1.07 | 1.00 | 0.18 | 1.23 | 1.41 | 0.62 | 1.32 |
